# Comparative Transcriptional Profiling Provides Insights into the Evolution and Development of the Zygomorphic Flower of *Vicia sativa* (Papilionoideae)

**DOI:** 10.1371/journal.pone.0057338

**Published:** 2013-02-21

**Authors:** Zhipeng Liu, Lichao Ma, Zhibiao Nan, Yanrong Wang

**Affiliations:** State Key Laboratory of Grassland Agro-ecosystems, School of Pastoral Agricultural Science and Technology, Lanzhou University, Lanzhou, China; Institute of Botany, Chinese Academy of Sciences, China

## Abstract

**Background:**

* Vicia sativa* (the common vetch) possesses a predominant zygomorphic flower and belongs to the subfamily Papilionoideae, which is related to *Arabidopsis thaliana* in the eurosid II clade of the core eudicots. Each vetch flower consists of 21 concentrically arranged organs: the outermost five sepals, then five petals and ten stamens, and a single carpel in the center.

**Methodology/Principal Findings:**

We explored the floral transcriptome to examine a genome-scale genetic model of the zygomorphic flower of vetch. mRNA was obtained from an equal mixture of six floral organs, leaves and roots. *De novo* assembly of the vetch transcriptome using Illumina paired-end technology produced 71,553 unigenes with an average length of 511 bp. We then compared the expression changes in the 71,553 unigenes in the eight independent organs through RNA-Seq Quantification analysis. We predominantly analyzed gene expression patterns specific to each floral organ and combinations of floral organs that corresponded to the traditional ABC model domains. Comparative analyses were performed in the floral transcriptomes of vetch and *Arabidopsis*, and genomes of vetch and *Medicago truncatula*.

**Conclusions/Significance:**

Our comparative analysis of vetch and *Arabidopsis* showed that the vetch flowers conform to a strict ABC model. We analyzed the evolution and expression of the TCP gene family in vetch at a whole-genome level, and several unigenes specific to three different vetch petals, which might offer some clues toward elucidating the molecular mechanisms underlying floral zygomorphy. Our results provide the first insights into the genome-scale molecular regulatory network that controls the evolution and development of the zygomorphic flower in Papilionoideae.

## Introduction

The pollinator-driven morphological diversification of flowering plants is closely associated with changes in the number, expression levels and interactions of a number of functional transcription factors. Studies in two core eudicot species, *Arabidopsis thaliana* and *Antirrhinum majus*, have led to the development of the classic ABC genetic model, which explains how three classes of genes (A, B, and C) interact spatially to specify floral organ identity [1], [2]. In *Arabidopsis*, A (*AP1*, *AP2*) determines sepals; A and B (*AP3*, *PI*) specify petals; B and C (*AG*) specify stamens; and C determines the carpel. Transcription expression profiling provides broader and deeper insight by extending beyond these key transcription factors commonly identified via mutant analyses to the genes downstream of the transcription factors that are involved in related pathways or networks, which may ultimately explain a given process. Using microarray techniques, this approach has been applied in the following angiosperms: the core eudicot *Arabidopsis*, at a whole-genome level [3]; the basal eudicots California poppy (*Eschscholzia californica*), using 6,446 ESTs [4], and *Aquilegia formosa*, using 17,246 unigenes [5]; and the basal angiosperms avocado (*Persea americana*), with 6,068 ESTs [6], and water lily (*Nuphar advena*), with 6,220 ESTs [7]. All other angiosperms are phylogenetically distant from model plants species (e.g., *Arabidopsis*), and have been analyzed using small or middle transcriptome. Therefore, previous studies might have been unable to recover transcriptome characteristics at a whole-genome scale due to the limited data available. Additionally, because only *Arabidopsis* belongs to the core eudicots, previous studies have tended to miss signatures of floral development and evolution within the core eudicots.

The Papilionoideae, most of which (28 tribes) exhibit specialized zygomorphic flowers, are distantly related to *Arabidopsis* in the eurosid II clade of core eudicots [8], [9]. This subfamily includes 30 tribes, 455 genera and approximately 12,000 species. Since the 1850s, the unique shape and the zygomorphic papilionoid flowers has been the subject of intense research, both due to its importance in Mendel’s groundbreaking work regarding genetic laws and as a model for studying organ differentiation and morphogenesis [9]–[23]. Papilionoid flowers differ greatly from those of other well-studied eudicots in terms of their floral structure, with most of these flowers possessing five sepals, five petals, ten stamens in two whorls and a single carpel. The organs within each whorl are initiated unidirectionally from the abaxial to the adaxial side. Additionally, in contrast to the strict timing order observed in *Arabidopsis*, the developmental timing of one whorl may overlap with that of the next whorl in some papilionoids [9], [13]. Recently, resources for transcriptome atlases have been developed in Papilionoideae, including *Lotus japonicus* [24], *Medicago truncatula* [25] and *Glycine max* [26]. In these studies, the flower was sampled as a whole organ without dissection to investigate gene expression. Therefore, a gap remains in the available datasets that would allow description the genome-wide transcriptional expression patterns in Papilionoideae flowers in terms of individual organs, such as the sepals, dorsal petals, lateral petals, ventral petals, stamens, and carpels.

Recent RNA deep sequencing technologies, such as Illumina transcriptome assembly analysis and RNA-Seq Quantification analysis, provide new opportunities to unravel the possible depths of transcriptome sequencing, even for species without a sequenced genome. Transcriptome assembly analysis generates millions of short cDNA reads, providing short, overlapping fragments that cover the entire transcriptome. RNA-Seq Quantification analysis provides a digital measure of the presence and prevalence of transcripts from known genes. The combination of these two approaches generates quantitative gene expression data and overcomes many of the inherent limitations of microarray-based systems [27]–[29].

Here, we have developed the first transcriptome expression analysis for individual floral organs in Papilionoideae using Illumina assembly technology and RNA-Seq Quantification analysis. The common vetch (*Vicia sativa* L.) belongs to the Papilionoideae and is a member of the Fabales clade, a sister clade to eurosid II (*Arabidopsis*) in phylogenetic reconstructions of the Rosids. We investigated the gene expression levels in the sepals, dorsal petals, lateral petals, ventral petals, stamens and carpels relative to the leaves and roots in *V. sativa* to provide an assessment of the spatial gene expression patterns in vetch flowers. Based on comparative analyses using data from a similar flower stage in *Arabidopsis* from a publically available dataset [3], we present insights into the general and unique molecular signatures of the evolution and development of the zygomorphic flower of the Papilionoideae at a whole-genome scale.

## Materials and Methods

### Plant materials and RNA extraction

**Figure 1:**
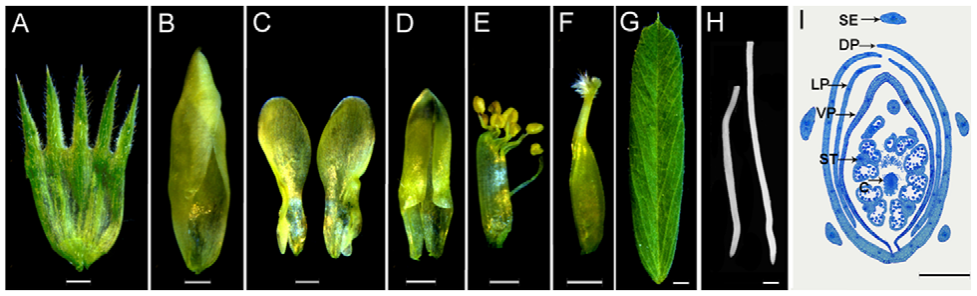
Eight organs isolated from vetch. (**a**–**i**) Sepal, dorsal petal, lateral petal, ventral petal, stamen, carpel, leaf, root and transverse section (2 µm) of a vetch flower at late pre-anthesis, which is identical to stage 12 in *Arabidopsis* [30]. C, carpel; DP, dorsal petal; LP, lateral petal; SE, sepal; ST, stamen; VP, ventral petal. Scale bars = 1.0 mm in (**a**–**g**). 5.0 mm in (**h**) and 500 µm in (**i**).

In this study, eight organs from the common vetch cultivar Lanjian 3 were investigated: the sepals, dorsal petals, lateral petals, ventral petals, stamens, carpels, leaves, and roots (Figure 1). Leaves and roots were collected from 2-week-old seedlings. The other organs were harvested from plants grown at Lanzhou University in Lanzhou, China for approximately 45 days in a greenhouse under a 16 hr light/8 hr dark cycle at 22°C. A total of 300 flowers were harvested at late pre-anthesis, from 100 individual plants and dissected into the six floral organs (sepals, dorsal petals, lateral petals, ventral petals, stamens, and carpels). The floral stage of vetch was identical to stage 12 in *Arabidopsis* [30]. All of the tissues were harvested between 9:00–12:00 a.m., placed immediately in liquid N_2_ and stored at −70 °C until RNA was extracted. Total RNA was extracted from the eight organs using the RNeasy Plant Mini Kit (Qiagen, Cat. #74904). RNA quantity and quality were assessed using a NanoDrop ND1000 instrument (Thermo Scientific).

### Light microscopy

Bright-field photographs of individual floral organs were obtained using a dissecting microscope (Leica, M205). Vetch flowers at late pre-anthesis were fixed in FAA (10% formalin, 50% ethanol, and 5% acetic acid) and dehydrated in a series graded ethanol. For histological analysis, the tissues were infiltrated with xylene and embedded in cold-polymerizing resin (Technovit 7100). The materials were then sectioned into 2 µm thick sections, stained with toluidine blue (Bio Basic) and observed using a light microscope. Transverse sections were photographed using a Nikon E600 microscope and a Nikon DXM1200 digital camera.

### Illumina sequencing, *de novo* assembly and functional annotation

For Illumina sequencing, equivalent quantities of total RNA isolated from the eight tissues were pooled. After poly(A) mRNA was purified and fragmented into small pieces, we used random hexamer primers and reverse transcriptase (Invitrogen) to carry out first-strand cDNA synthesis. Second-strand cDNA synthesis was performed with RNase H (Invitrogen) and DNA polymerase I (New England BioLabs). We constructed a cDNA library with average insert sizes of 200–500 bp and conducted cDNA sequencing using the Illumina HiSeq™ 2000 system according to the manufacturer’s protocols, with a read length of 100 bp. The average proportion of clean reads for the library was 96.8%. The transcriptome sequence was assembled into distinct contigs using the short reads with SOAPdenovo software [31] (http://soap.genomics.org.cn). The paired-end relationships between the reads were employed to construct scaffolds between the contigs. We next filled the intra-scaffold gaps and constructed a non-redundant unigene set from all three of the assembled datasets using the EST assembly program TGICL [32]. We annotated the sequences based on protein databases, such as nr, Swiss-Prot, KEGG, and COG (E-value <10E-5) by retrieving the proteins with the highest sequence similarity to the given unigenes, along with their functional protein annotations. The Blast2GO program [33] was employed to obtain GO annotations for the unigenes.

### RNA-Seq Quantification analysis

Eight independent cDNA libraries were constructed for the eight organs in parallel according to the RNA-Seq protocol. Raw image files were collected using the Illumina HiSeq™ 2000 sequencing platform in BGI Shenzhen (http://en.genomics.cn/navigation/index.action). The data analyzed have been deposited on the NCBI Gene Expression Omnibus under accession no. GSE35437.

### Quantitative RT-PCR

Total RNA was isolated from six floral organs as well as leaves and roots with the RNeasy Plant Mini Kit. For first-strand cDNA synthesis, we used 2.5 µg of total RNA and followed the manufacturer's protocol (TaKaRa Biotechnology, Dalian, China). The cDNA samples were diluted to a concentration of 2.5 ng/µl. Quantitative RT-PCR (qRT-PCR) was performed in triplicate using the 7500 Real-time PCR System (Applied Biosystems) with 4 µl of the cDNA dilution and Power SYBR Green Master mix (Applied Biosystems), according to the manufacturer's protocol. Unigene68614, which has an unknown function, was used as an internal control because of its relatively invariable expression across the transcription profiles. The mRNA fold changes in the different samples were calculated by the 2^ΔΔCt^ method, as described previously [34]. In a semi-quantitative PCR analysis to confirm the sequencing data, we employed the same primers as for the qRT-PCR.

### Statistical analyses

The expression level of each gene was measured via RNA-Seq Quantification analysis as the number of reads per kilobase of an exon region in a given gene per million mapped reads (RPKM) [35]. To identify differentially expressed unigenes among the eight organs, the Z-score transformation normalization method was applied to compare the expression levels of the unigenes from the floral organs, leaves and roots and to directly calculate the differences in unigene expression levels between the different samples [36], [37]. Z-scores were calculated by dividing the difference between the target organ or combination of organs (Xi, organ or mean of organs) and all of the other organs (µ, mean) by the standard deviation (SD) of all of the other organs using the following equation:
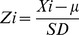



Additionally, we measured the relative Ratio among samples using the following equation:



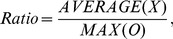



where *X* represents the expression level in the target organ or combination of organs, and *O* is the expression level in all of the other organs. The expression levels in both the common vetch and *Arabidopsis* were analyzed using the above equations.

### Comparative floral transcriptome

To compare the evolutionary patterns of the floral transcriptomes, we compared the floral expression patterns of vetch and *Arabidopsis*. Microarray expression data for the leaves, roots and floral organs of stage 12 in *Arabidopsis* were extracted from datasets [3]. To provide new insights into the development of the dorsal petal, we performed a homologous comparison between genomes of vetch and *M. truncatula*. The genome data *M. truncatula* were from Mt3.5 Release (http://tofu.cfans.umn.edu/). Based on nucleotide sequence similarity, a reciprocal BLAST search was performed with a cutoff E-value<10E-5 using blast-2.2.23-ia32-win32. The expression data were visualized using MeV v4.6 (http://www.tm4.org/mev/).

### Gene Ontology analyses and *cis*-elements analyses

The molecular function and cellular component term "enrichment status" and "hierarchy" of unigenes were analyzed via agriGO software (http://bioinfo.cau.edu.cn/agriGO/analysis.php) [38]. The PLACE database (http://www.dna.affrc.go.jp/PLACE/) [39] was used for *cis*-element search in the promoter regions of homologous genes in *M. truncatula*. We selected an approximate 2,000 bp promoter region in each gene to search for the possible *cis*-element.

## Results

### Transcriptome characteristics in floral organs

After using paired-end information to join contigs into scaffolds and local assembly, we generated a total of 71,553 high-quality unigene sequences with lengths greater than 200 bp, and the average length of the unigenes was 511 bp. The size distribution of the assembly is displayed in Figure S1. All unigene sets were then annotated based on similarities to known or putative sequences available in public databases. The COG function classification of the vetch unigenes is displayed in Figure S2.

Using 71,553 unigenes, we analyzed the genome-scale gene expression profiles in eight organs, i.e., the sepals, dorsal petals, lateral petals, ventral petals, stamens, and carpels, using the leaves and roots as controls (Figure 1). The qRT-PCR analysis and semi-quantitative PCR analysis were used to confirm the sequencing results (Figure S3A and Table S1). A total of 60 unigenes displaying biased or unbiased expression in the RNA-Seq Quantification analysis was examined (Table S1). The Illumina sequencing and qRT-PCR results displayed a significant positive correlation for 49 of the 60 unigenes (R>0.707, P<0.05), which demonstrated that approximately 81.7% of the Illumina sequencing data could be directly validated using qRT-PCR. Furthermore, the semi-quantitative PCR results (Figure S3B) correlated well with the expression patterns of the 49 unigenes detected via Illumina sequencing.

Additionally, based on the unigene expression values obtained for each organ, we analyzed the Spearman correlation coefficients (r-value) between the pairs of organs (Table S2A) and obtained the highest correlation coefficients for the pairs of the three types of petals: dorsal petals/lateral petals (0.9399), lateral petals/ventral petals (0.8552) and dorsal petals/ventral petals (0.8119). The lowest correlation coefficient (0.2492) was observed when comparing the carpels/lateral petals. After combining the three kinds of vetch petals into a single category (the petals), the highest correlation coefficient (0.7197) was found between the petals and stamens in vetch (Table S2B). Hierarchical clustering of the total of 71,553 vetch unigenes sorted the floral organs into groups based on the similarity of the unigene expression patterns. The lateral and dorsal petals clustered together, followed by the ventral petals and the stamens, while the sepals and the carpels clustered distantly from the other floral organs (Figure S4).

### Identification of enriched and downregulated unigenes in the floral organs

We identified unigenes that were enriched in the floral organs by comparing the expression levels of the unigenes in individual floral organs and in other tissues with cutoffs of a Ratio >2.0 and Z-score >3.75 [36]. These analyses revealed that 9,502 unigenes were enriched in the individual floral organs in vetch. The carpels (4,011) exhibited the greatest number of the enriched unigenes (Table S3A). To achieve correspondence with the floral structure of *Arabidopsis*, we combined the three types of vetch petals into a single category and obtained 3,541 unigenes that were preferentially expressed in the petals. We used similar methods to screen for genes enriched in the floral organs of *Arabidopsis* [3] and determined the stamens possessed the greatest number of the enriched genes (Table S3A).

The genes that were downregulated in the floral organs showed a similar characteristic to the genes that were enriched in the floral organs. We chose parameters with a relatively low stringency (Ratio<0.5 and Z-score<−1.5) to screen for unigenes that were downregulated in the floral organs. We determined that 495 unigenes were downregulated in the sepals, 624 in the petals, 3,855 in the stamens and 12,504 in the carpels in vetch (Table S3B). Consistent with analysis of unigenes that were enriched in individual organs in vetch, the carpels also exhibited the greatest number of the downregulated unigenes. Using a similar method (Ratio<0.5 and Z-score<−1.5), we screened the genes that were downregulated in the floral organs of *Arabidopsis* [3] (Table S3B). Similar to our previous results in *Arabidopsis*, the stamens also displayed the greatest number of downregulated genes.

Furthermore, we analyzed the functional features of the enriched and downregulated transcripts in the investigated organs according to Gene Ontology terms in the transcriptome annotation for vetch (Figure S5 and Table S4). The enriched and downregulated unigenes in the floral organs of vetch were classified into 14 functional groups, and the distribution of these unigenes showed a clear variance among distinct floral organs (Figure S5). Both the enriched and the downregulated transcripts showed an obvious skew toward metabolic processes, cellular processes and responses to stimuli. However, the functional features of the enriched and the downregulated unigenes were strikingly different in some categories, such as cell wall organization or biogenesis, death-related transcripts, stimulus and death. These findings indicated that organ-dependent enrichment or downregulation of different unigenes is essential for floral organ differentiation, development, and functional specification.

To further analyze the molecular regulation of floral organ development, we searched the differentially expressed unigenes that were both enriched in individual organs and enriched in three combinations of organs (representing the A, B, and C domains) using cutoffs of a Ratio>2.0 and Z-score>3.75. This screening identified 24,472 unigenes that were enriched in vetch floral organs (Table S3A): 9,502 displayed enrichment in individual organs, whereas 14,970 were enriched in a combination of organs. It is conceivable that the unigenes enriched in the individual organs and those enriched in the combinations of organs might play important roles or be involved in pathways downstream of the key regulators involved in floral organ development. Therefore, we focused on the expression characteristics of six functional groups (4,501 of the 24,472 unigenes enriched in the floral organs): transcription, phytohormones, reproduction, kinase systems, stimulus responses and signaling transduction (Table S5A).

For example, the unigenes belonging to 41 distinct transcription factor families displayed a heterogeneous distribution among the four individual organs and the three organ combinations. However, these 41 transcription factor families exhibited a significant skew for the following 13 families: MYB/MYB related, MADS, NAC, bHLH, C3HC4, WRKY, LIM, BTB/POZ, bZIP, GRAS, ARF, HMG and SBP (Table S5B). Among the single organ, the number of transcription factors in the carpels was the greatest, indicating that the development of the carpels may require more transcription factors compared to the other organs. This result also explained why the carpels possessed the greatest numbers of both enriched unigenes and downregulated unigenes among the vetch floral organs (Tables S3A and S3C). The enriched and downregulated unigenes might be the downstream target genes of the 13 transcription factor families. Additionally, a total of 1,385 unigenes that were enriched in individual organs or in a combination of organs encoded kinase domain-containing proteins (Table S5D). Consistent with calcium signaling playing an important role in pollen development, the calcium signal-related kinase unigenes were mainly distributed in the stamens, which were characterized by containing abundant mature pollen [30], [36].

### Comparative floral transcriptome analysis between vetch and *Arabidopsis*

We compared potentially conserved and diverse evolutionary features of the vetch and *Arabidopsis* floral transcriptomes based on published data for the transcriptomes of *Arabidopsis* flowers [3]. Using BLAST searches with a cutoff E-value<1.0E-05, we found that 23.6% (6,152) of the 26,194 unigenes (1,819 enriched unigenes in the three individual petal types subtracted from 28,013 total unigenes) enriched in individual organs or a combination of organs in vetch had homologous transcripts in the *Arabidopsis* genome. A total of 3,367 (56.4%) of these 6,152 homologous unigenes were expressed in individual organs or a combination of organs in *Arabidopsis*. Furthermore, 1,895 (27.5%) of these 6,152 unigenes were enriched in individual organs or a combination of organs in *Arabidopsis* (Tables 1, S6A, and S6B). Interestingly, among the individual organs, the unigenes enriched in the vetch stamens displayed the greatest number (473) of enriched homologs in *Arabidopsis* organs.

**Table 1:** Numbers of enriched unigenes in vetch and their homologs in *Arabidopsis*.

Expression pattern	Sepals	Petals	Stamens	Carpels	A domain genes	B domain genes	C domain genes	Total
EE	861	3541	2811	4011	3797	7798	3375	26194
NH	689	2933	2041	2590	3211	6198	2380	20042
DE	117	383	216	1058	390	696	507	3367
EE	17	97	473	184	88	712	324	1895
UE	38	128	81	179	108	192	164	890
HH*	172	608	770	1421	586	1600	995	6152

NH, no homolog; HH, having a homolog; DE, detectable expression; EE, enriched expression; UE, undetectable expression. The asterisk indicates that HH is comprised of HE, EE, and UE. Homologous comparison using an E-value<1.0E-05 and enriched expression analysis using the cutoffs of a Ratio>2.0 and Z-score>3.75. The *Arabidopsis* data were obtained from Schmid et al. [3].

In contrast, further BLAST reaches against the vetch database using the *Arabidopsis* sequences determined that 3,496 (56.1%) of the 6,412 genes enriched in individual organs or a combination of organs in *Arabidopsis* exhibited homologous transcripts in the vetch genome. A total of 1,890 (58.1%) of these 3,496 homologous genes were expressed in individual organs or a combination of organs in vetch, whereas 1,291 (31.6%) of these 3,496 genes displayed enriched expression in individual organs or a combination of organs in vetch (Tables S6A and S6C). Consistent with the results described above, the stamens (365) also presented the greatest number of enriched homologs in the two plants, which might suggest the existence of non-conserved expression patterns in the counterpart floral organs of vetch and *Arabidopsis*, with the exception of the stamens.

Additionally, we compared the expression patterns of the homologous transcripts that were enriched in both vetch and *Arabidopsis*. Only 1,052 unigenes (after deleting duplicates from 1,895 unigenes) displayed enrichment in counterpart organs in both vetch and *Arabidopsis*; these transcripts were mainly involved in hydrolase activity, transcription factor activity, transferase activity, protein binding, DNA or RNA binding, and kinase activity.

**Figure 2:**
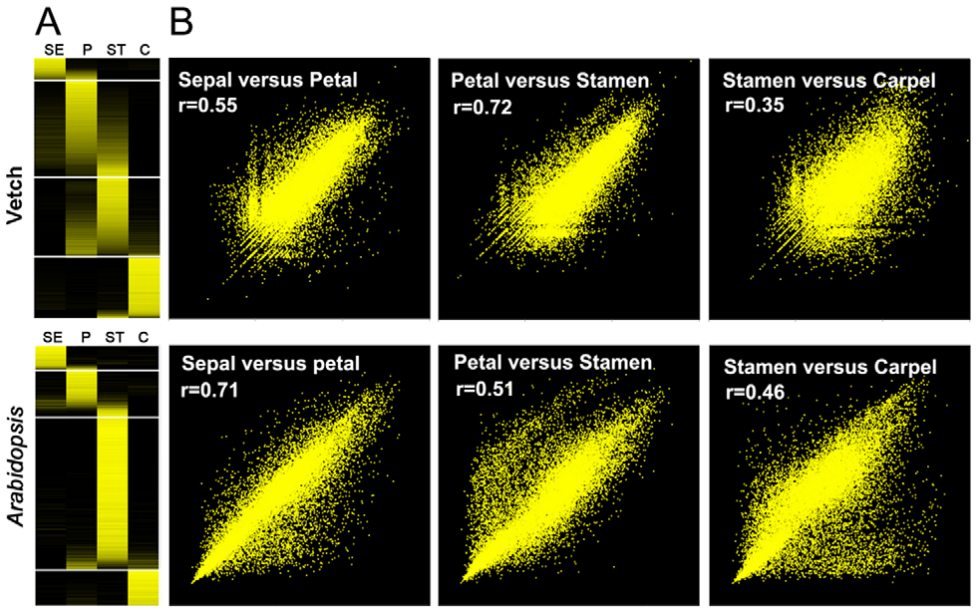
Floral organ transcription patterns in vetch and *Arabidopsis*. (**a**) Log2 floral organ/non-adjacent-organ gene expression ratios in vetch and *Arabidopsis*. Transcripts showed enriched expression in floral organs in vetch and *Arabidopsis* using cutoffs of a Ratio>2.0 and Z-score>3.75. C, Carpel; P, petal; SE, sepal; ST, stamen. The scale of the log2 ratios ranges from −1.5 (black) to 1.5 (yellow). The analyses are based on 3,903 *Arabidopsis* and 20,714 vetch transcripts enriched in the floral organs. (**b**) Scatter plots and Pearson correlations (r) of the gene expression levels in adjacent floral organs in vetch and *Arabidopsis*. The analyses are based on all of the *Arabidopsis* genes (21,972) and all of the vetch unigenes (71,553). The *Arabidopsis* data were obtained from Schmid et al. [3].

We further compared the spatial distribution of floral-biased expression in vetch and *Arabidopsis* flowers [40]. Using log2 floral organ/non-adjacent-organ gene expression values (Ratio>2.0 and Z-score>3.75), we obtained a total of 3,903 *Arabidopsis* transcripts and 20,714 vetch unigenes enriched in the floral organs. Similar to the results for *Arabidopsis* [40], the enriched vetch transcripts demonstrated sharp boundaries among the different floral organs (Figure 2A). Based on all of the transcript expression data for *Arabidopsis* (21,972 genes) and vetch (71,553 unigenes), we constructed scatter plots for the adjacent floral organ categories. Among the floral organwise categories, the Pearson correlations were 0.55, 0.72, and 0.35 for vetch, and 0.71, 0.51, and 0.46 for *Arabidopsis* (Figure 2B). These results illustrated that the adjacent floral organs are divergent from each other in both species, suggesting that vetch flowers may be shaped following a strict ABC model without a “fading border” [40].

Furthermore, we examined transcription factors, which are key regulators of the events that involve in organ development to compare the conserved features of the transcriptomes of the two species. Among the 655 transcription factors unigenes enriched in the vetch floral organs (Table S6), 120 homologs from 16 transcription factor families were found to also display enriched expression in *Arabidopsis* organs (with an E-value<1.0E-05), including the MYB/MYB related, NAC, MADS and HOMEOBOX families, which suggested that these transcription factors share conserved functions in vetch and *Arabidopsis*. Interestingly, 102 unigenes from 17 transcription factor families, such as C4HC4, LIM, TLP, SWI/SNF, HSF, CCAAT, and AS2, appeared to be specific to vetch flowers, and none of these famlies were enriched in *Arabidopsis*. However, eight genes from six transcription factor families, including DDT, VP1/ABI3, and S1FA-like, that displayed enriched expression in *Arabidopsis* were not enriched in vetch.

**Figure 3:**
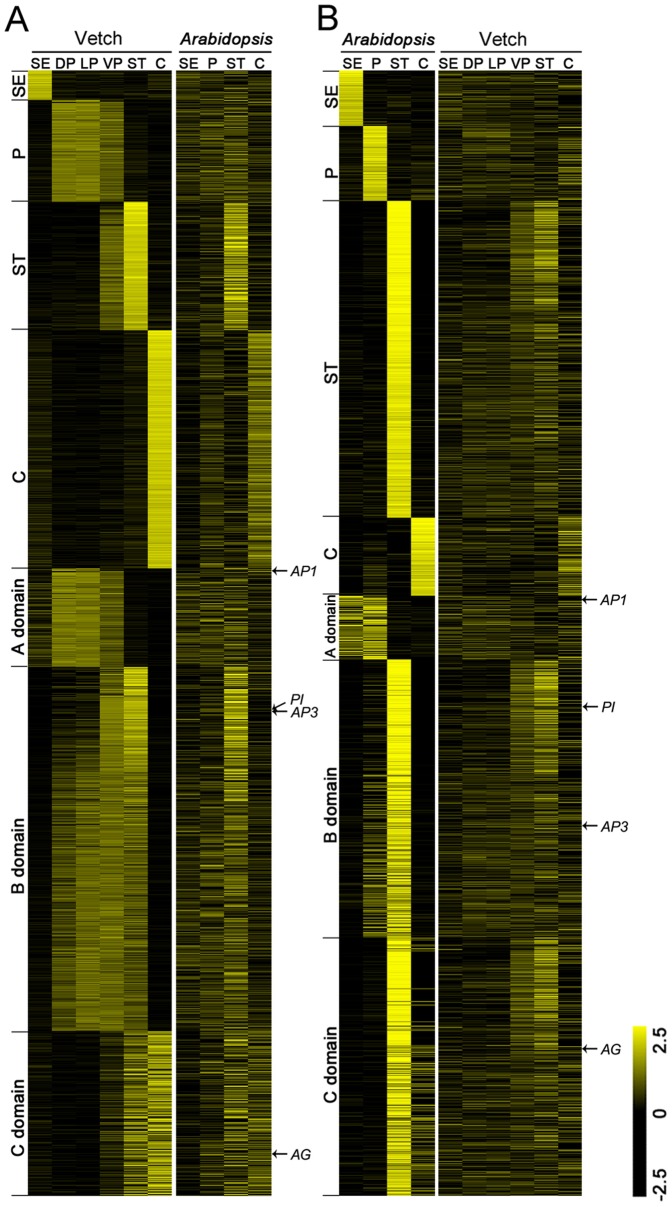
Expression patterns of the homologous transcripts identified between vetch and *Arabidopsis* organs. (**a**) Expression patterns of the unigenes (5,891) enriched in vetch and their homologs (arranged in the same row) in *Arabidopsis* (E-value<1.0E-05). (**b**) Expression patterns of the genes (3,367) enriched in *Arabidopsis* and their homologs in vetch (E-value<1.0E-05). The bar represents the scale of the expression levels of the transcripts. The scale of the log2 ratios ranges from −2.5 (black) to 2.5 (yellow). The abbreviations for the different tissues are the same as in Figure 1. The *Arabidopsis* data were obtained from Schmid et al. [3].

We further compared the spatial expression profiles of one-to-one homologous enriched transcripts in vetch and *Arabidopsis* (Figure 3 and Table S7). Compared with the organ-enriched unigenes (6,152) in vetch, most of the *Arabidopsis* homologs skewed toward equivalent expression among the different organs, whereas only a few homologs in *Arabidopsis*, such as a subset of genes in the stamens and the carpels, displayed enriched expression patterns (Figure 3A). The genes in the C domain exhibited relatively conservative expression patterns between the two species. In contrast, most of the homologs of the genes (3,496) enriched *Arabidopsis* showed unbiased expression patterns in vetch, except for the genes enriched in the stamens and the carpels (Figure 3B). These results suggested that the expression patterns of downstream homologs in the two species display weak conservation among the floral organs, despite the fact that vetch and *Arabidopsis* both belong to the Rosids in the core eudicots.

### Floral zygomorphy of vetch revealed by transcriptional profiling

**Figure 4:**
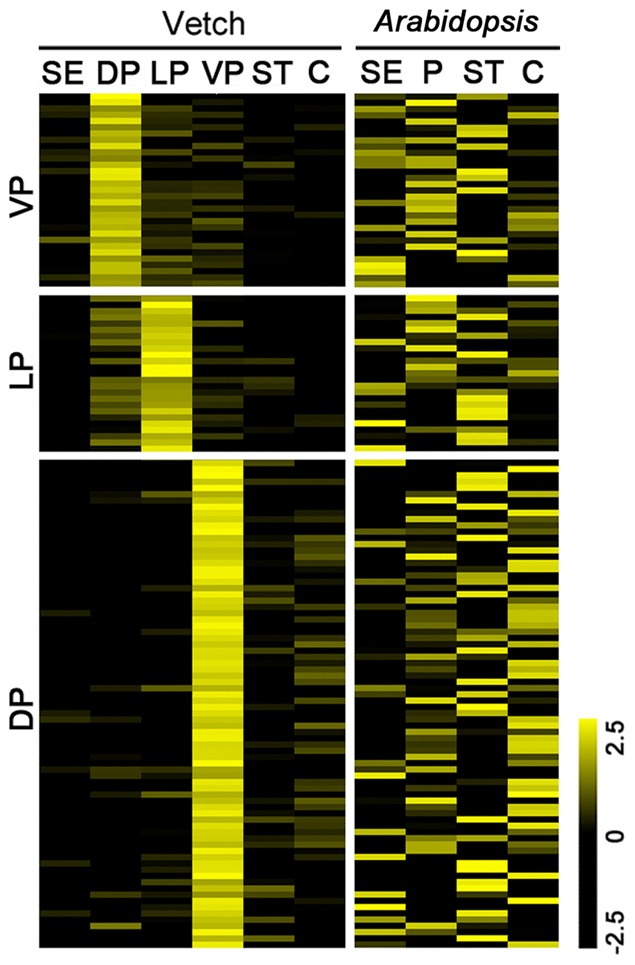
Expression patterns of homologous transcripts between vetch and *Arabidopsis* petals. The unigenes enriched in the three types of petals and their homologs in *Arabidopsis* (E-value<1.0E-05) are arranged in the same row. Most of the *Arabidopsis* homologs display equivalent expression among the floral organs. The bar represents the scale of the expression levels of the transcripts. The scale of the log2 ratios ranges from −2.5 (black) to 2.5 (yellow). The abbreviations for the different tissues are the same as in Figure 1. The *Arabidopsis* data were obtained from Schmid et al. [3].

We first identified the unigenes that were preferentially expressed in the three types of the vetch petals using cutoffs of a Ratio>2.0 and Z-Score>3.75. We then performed BLAST searches against the *Arabidopsis* database using a cutoff E-value<1.0E-05. A total of 31, 25, and 80 unigenes showing specific expression in the dorsal, lateral and ventral petals, respectively, were found to have homologous transcripts in the *Arabidopsis* genome (Figure 4). Among the unigenes presenting specific expression patterns in the three different types of vetch petals, most of the *Arabidopsis* homologs displayed equivalent expression levels among the different organs. The unigenes that are specifically expressed in vetch petals may contribute or respond to morphological differences in the petals between vetch and *Arabidopsis*. To provide fresh insights into the transcription regulation of gene expression in the dorsal petal development, we performed a homologous comparison via BLAST searches (E-value<1.0E-05) against the *M. truncatula* genome database using 462 transcripts specific in vetch dorsal petals. A total of 100 homologous genes were obtained from *M. truncatula*. We then researched *cis*-acting regulatory elements from approximate 2,000 bp regions upstream of start codon using the PLACE database. Of 237 identified *cis*-elements, the five highest binding sites were YACT, AAAG, CAAT, GRWAAW, and NGATT (Table S8A). Impressively, 104 putative CYC or TCP binding sites GGNCCC were identified in 49 genes in *M. truncatula* (Table S8B). The gene *AC233140_70.1*, also named *CYC1A*, was included [23]. Additionally, analyses of the 462 dorsal petal specific unigenes in biological process and molecular functional terms "enrichment status" and "hierarchy" showed that ion transmembrane transport and localization had significant importance in the development of the dorsal petals (Figure S6A and S6B). Transcripts involved in internal side of plasma membrane seemed be more important in the dorsal petal specific data (Figure S6C).

**Figure 5:**
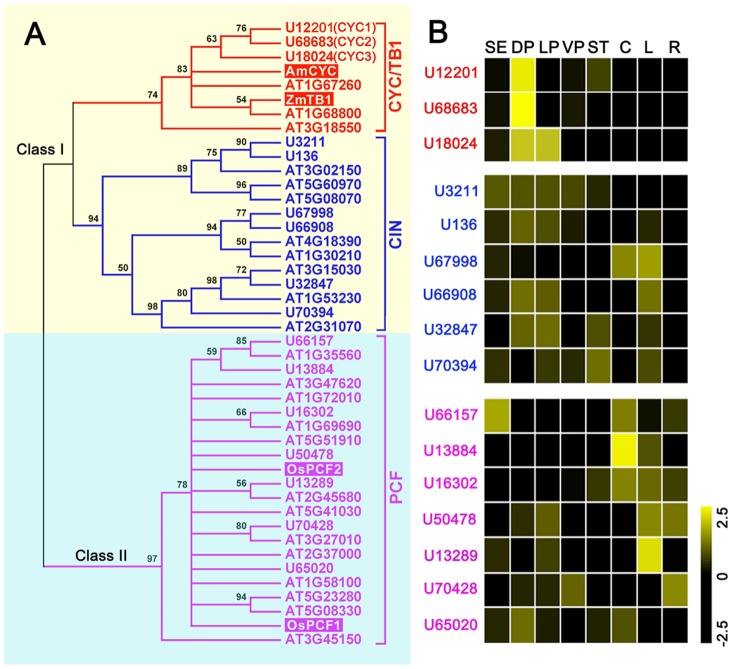
Neighbor-joining consensus tree and expression patterns of TCP genes. (**a**) Unrooted protein tree for the TCP protein domain data set produced using MEGA 4.0.1 [73]. The tree summarizes the evolutionary relationships among the 24 *AtTCPs* [41], 16 *VsTCPs*, and other representative sequences. The bootstrap values are denoted above the nodes; bootstrap values of less than 50 are not shown on the phylogenetic tree. The representative sequences include *OsPCF1* (NP_001052212) and *OsPCF2* (NP_001062409) in rice; *ZmTB1* (AF377742) in maize; and *AmCYC* (Y16313) in snapdragon. (**b**) The expression patterns of the TCP genes in vetch organs. The abbreviations for the different tissues are the same as in Figure 1.

Transcription factors in the TCP (*TEOSINTE BRANCHED1* from maize, *CYCLOIDEA* from snapdragon, and *PCF* from rice) family regulate cell division in vegetative and reproductive structures and are essential for establishing flower zygomorphy. To understand the evolutionary relationship between the TCP genes of vetch and *Arabidopsis*, we identified 16 and 24 TCP genes in vetch and *Arabidopsis*, respectively [41]. Through performing a phylogenetic analysis of the TCP protein domain, we grouped these TCP genes into three clades: PCF, CYC/TB1, and CIN (Figure 5A). There were seven unigenes in the PCF clade, three unigenes in the CYC/TB1 clade and six unigenes in the CIN clade. In the NJ phylogenetic tree, we observed seven well-supported clades containing both vetch and *Arabidopsis* genes, suggesting that the most recent common ancestor of vetch and *Arabidopsis* had at least seven TCP genes. Because there are a few additional clades including only vetch or *Arabidopsis* genes, the number of TCP genes in the most recent common ancestor was probably greater than seven. Furthermore, the expression pattern of the TCP genes in vetch was analyzed, and the genes in the CYC/TB1 clade were preferentially expressed in vetch petals, while the genes in the PCF and CIN clades exhibited unbiased expression among the eight vetch organs (Figure 5B). This interesting result might indicate that only the CYC/TB1 genes in the TCP family are involved in floral zygomorphy in vetch.

## Discussion

### Genome-scale gene expression patterns in vetch

Based on *de novo* assembly and RNA-Seq Quantification analysis, our examination of the genome-scale gene expression profiles in eight organs in vetch revealed characteristics of the transcriptomes of the floral organs and provided novel insights into the evolution and development of the zygomorphic flower. Comparative transcriptional analyses of wild-type and mutant flowers of *Arabidopsis* have distinguished a large number of genes that are not expressed in ABC gene mutants and are likely downstream of these key transcription factors [42], [43]. In this study, we identified unigenes enriched in individual floral organs and combinations of organs in vetch. The unigenes enriched in the different organ combinations may be regulated by and associated with the homologs of *AP1*, *AP3*, *PI*, *AG*, and *SEP1* in vetch. For the *AP1* homolog, expression peaks were mainly detected in the sepals and the dorsal, lateral, ventral petals (Figure S7). Vetch homologs of *AP3* and *PI* were strongly expressed at late pre-anthesis in the three types of petals and the stamens, similar to the pervious findings that persistent activity of *AP3* and *PI* through the late stages of petal development was necessary to maintain the expression of characteristic petal features [44]–[46]. Additionally, the *AG* homolog was highly expressed in the stamens and carpels, while expression of *SEP1* homolog was observed throughout the vetch flower. Therefore, our findings related to the ABC regulators in vetch were consistent with what is observed in other angiosperms, such as *Arabidopsis* [1], [2], *L. japonicus* [16], *Petunia hybrida* [47], *Oryza sativa* [48]–[50], *Gerbera hybrida* [51], *Zea mays* [52], *Pisum sativum* [53] and *Eschscholzia* [4].

Among the A domain unigenes (Figure 3), all of the transcripts were strongly expressed in the three types of petals, whereas the expression levels were not as high in the sepals. Considering the dubious role of a “true” A function in the control of perianth identity [54]–[56], our results have provided a few valuable clues for future molecular studies. The B domain genes, *AP3* and *PI*, are best understood from an evolutionary standpoint, and these genes appear to be associated with a gene duplication event that occurred prior to the radiation of angiosperms [57]. Although the B domain unigenes were found to be expressed at higher levels in the petals and stamens in the present study, there were apparently also some organ-specific transcripts, especially in the three different petal types (Figure 3), which is much different from what is observed in *Arabidopsis* and may be important for the development of petals in vetch. It was therefore tempting to speculate that the B domain genes of the Papilionoideae have undergone gene duplication and differentiation, leading to the genes controlling the identity and development of the three complex types of petals following the radiation of the B domain genes in angiosperms.

Interestingly, when the relationships among the organs were examined based on global transcription expression levels (Figure S4), the three types of vetch petals clustered tightly with the stamens. We provided further support for this finding by calculating the Spearman correlation coefficients between pairs of vetch organs (Table S2). The results indicated that the dorsal, lateral, and ventral petals shared some common genetic programs and were process homologous. Also, this finding is in agreement with the traditional hypothesis of the petals morphologically originating from the stamens in core eudicots [58], [59]. The clustering of the petals with the stamens in vetch might be explained by the following findings. An interesting characteristic is provided by the description of the common primordia in some Papilionoideae flowers from which the petals and the stamens differentiate [9], [12], [13], [60].

### Characteristics of the floral transcriptomes of vetch and *Arabidopsis*

*Arabidopsis* is the best-characterized experimental model among the eudicot plants, and global gene expression data for specific floral organs are available for this species. Our analyses suggested that there are both conserved and divergent features of the floral transcriptomes of vetch and *Arabidopsis*. Homologs showing similar enriched expression patterns in the two species were mostly found to be involved in basic processes, such as hydrolase activity, transcription factor activity, transferase activity, protein binding, DNA or RNA binding, and kinase activity. The expression of transcription factors was conserved between the two species for the MYB/MYB-related, NAC, MADS, and HOMEOBOX families. Additionally, consistent with other angiosperms, homologous ABC regulator genes exhibited similar expression patterns in the two species (Figure 3), suggesting that major components of their functions may be conserved throughout angiosperm evolution [7]. The genes participating in the transcriptional programs of vetch and *Arabidopsis* flowers were tightly constrained to individual floral organs (Figure 2A), and organwise comparisons between adjacent floral organ categories showed similar correlations in the two species. Therefore, we concluded that vetch flowers display strict spatial ABC domains and sharp transcriptional boundaries among the floral organ categories, as observed in *Arabidopsis* flowers. A similar result was established in *Eschscholzia* [4], [40], suggesting that the flowers of more recently derived angiosperm lineages with highly differentiated perianths, such as those of the core eudicots (represented by vetch and *Arabidopsis*) and some basal eudicots (represented by *Eschscholzia*), are more likely to be characterized by a strict ABC model with sharp transcriptional boundaries among adjacent floral organ categories, despite the “fading borders” observed in other basal angiosperms (represented by *Aquilegia*, because of its distinctive floral anatomy) [5].

The percentage of transcripts enriched in vetch sepals (1.20%) is similar to that in *Arabidopsis* sepals (1.33%), implying that vetch tends to share a similar evolutionary scenario with *Arabidopsis* in terms of sepal development (Table S3A). With respect to the different categories of petals, the greater number of transcripts enriched in vetch (3,541) compared with the number of genes enriched in *Arabidopsis* (368) suggested that the petal developmental program in vetch is more complex than that of in *Arabidopsis*. Moreover, the genes showing enriched expression in vetch petals displayed a broader expression domain (4.95%) than is observed in *Arabidopsis* petals (1.67%) (Table S3A). Considering the three highly differentiated types of petals observed in vetch, this result can be explained by the greater complexity of the petal organ category in vetch versus *Arabidopsis*. Additionally, the vetch stamens and carpels displayed a larger number of enriched unigenes, which is consistent with the model eudicot *Arabidopsis*, and the basal angiosperms, *Nuphar* and *Persea* [7]. This conservation of transcript numbers might be explained by the diversity of cellular events taking place in the stamens and carpels. In vetch, the carpels displayed the largest number of enriched unigenes and the most downregulated unigenes, whereas the stamens presented the greatest number of enriched and downregulated unigenes in *Arabidopsis*. This finding suggested possible differences between stamen and carpel development in the two species. Furthermore, we analyzed the evolutionary conservation of the transcripts enriched in vetch and *Arabidopsis* based on the expression patterns of their homologous transcripts (Figure 3). Compared to the sepals and petals, the homologs of the transcripts enriched in the stamens and the carpels exhibited relatively more highly conserved expression patterns in vetch and *Arabidopsis*, suggesting conservation of gene expression between the two species in the molecular regulation of stamen and carpel development.

Because both vetch and *Arabidopsis* display strict spatial ABC domains, we compared the spatial expression patterns of one-to-one homologous transcripts between vetch and *Arabidopsis* (Figure 3). It was determined that the downstream transcriptional regulatory machinery responding to the ABC domains in the two species was not conserved with regard to the expression patterns of the genes involved. With respect to the genes displaying enriched expression in the floral organs of vetch, a high percentage of homologs did not show enriched expression in the counterpart organs of *Arabidopsis* and vice versa (Table 1 and Table S7). Our results were supported by a previous study in which it was determined that most of the genes displaying enrichment in *Arabidopsis* floral organs were downregulated in *Nuphar* and *Persea*, whereas those showing enrichment in *Nuphar*, *Persea*, or both, were downregulated in *Arabidopsis*. Recent large-scale studies of evolutionary changes in gene expression among several non-plant species have led to the proposal that there is a substantial amount of divergence in orthologous gene expression [61]–[65]. However, the divergence of orthologous gene expression levels is significantly lower for pairs of randomly chosen genes. Furthermore, divergence in the expression of essential genes appears to be strongly constrained by natural selection, and other genes tend to be under minimal selective constraints [64]. Therefore, we assumed that the key regulatory genes involved in the ABC domains in eudicot plants are likely subjected to stronger selection and display a conserved expression pattern among diverse floral organs. A large proportion of downstream orthologous transcripts in ABC domains have been subjected to minimal selection and display divergence in spatial expression during flower evolution and diversification.

### Control of floral zygomorphy in vetch

One of the most distinct aspects of Papilionoideae species is their zygomorphy, which originated indendently several times from actinomorphic ancentors (showing radial floral symmetry) and subsequently expanded to many large and successful angiosperm lineages [19], [23]. Vetch flowers, like most zygomorphic flowers, possess two types of asymmetries: dorsoventral asymmetry in the floral plane and organ internal asymmetry in the floral organ plane. The internal asymmetry is different among the various petals: the dorsal petal is internally symmetric, and two lateral petals and two ventral petals are internally asymmetric. In *P. sativum* and *L. japonicus*, the floral zygomorphy depends on the activity of the TCP transcription factors. *CYC2* plays a role in establishing the identity of the dorsal petals, while *CYC3* plays a key role in controlling the identity of the lateral petals [19], [21]. Both *CYC1* and *CYC2* are specifically expressed in the dorsal petals to promote the growth of the single dorsal petal, while *CYC3* is strongly expressed in the dorsal and lateral petals [19], [21]. In the present study, the expression patterns of *VsCYC1*, *VsCYC2*, and *VsCYC3* in vetch were found to be similar to their patterns in other species (Figure S7), which was reconfirmed using qRT-PCR and semi-quantitative PCR (Figure S3 and Table S1). This finding suggested that the three *VsCYC* genes might also be crucial in controlling floral zygomorphy in vetch.

A pair of dorsal-specific *CYC* genes is usually redundantly or partially redundantly involved in the establishment of floral zygomorphy, with one of the copies exhibiting no or transient expression in floral zygomorphic regions, such as the *CYC* and *DICH* genes in snapdragon [66], [67], *CYC1* and *CYC2* in *Mohavea confertiflora* [68], *CYC1A* and *CYC1B* in *Lupinus nanus* (Fabaceae) [17], *CYC1* and *CYC2* in *L. japonicus* [19], *CYC1* and *CYC2* in pea [21], *CYC1C* and *CYC2A* in *Opithandra dinghushanensis* [69], *CYC2A* and *CYC2B* in *Byrsonima crassifolia* (Malpighiaceae) [70], *CYC2A* and *CYC2B* in *Lonicera* (Caprifoliaceae) [71], and *CYC1C* and *CYC1D* in *Primulina heterotricha* [23]. Furthermore, it seems very likely that a double positive autoregulatory feedback loops have been employed by *CYC* genes in different zygomorphic clades, such as in *M. truncatula* and *G. max* [23]. Therefore, the expression of *VsCYC1* and *VsCYC2* might be sufficient to generate morphological zygomorphy in vetch, and these genes may be regulated by the double positive autoregulatory loops, which is a possibility that deserves further research.

Because the three vetch *CYC* genes are members of the CYC/TB1 clade in the TCP family, we further examined the expression patterns of TCP family genes using genome-scale analysis to investigate the relationship of floral zygomorphy with the other TCP members in vetch. Based on amino acid sequence similarity in the TCP domain, the TCP family is divided into two major clades: Class I and Class II. The latter clade is further subdivided into two additional clades: the CYC/TB1 clade and the CIN clade [41]. Among the three clades, we found that only genes in the CYC/TB1 clade were preferentially expressed in vetch petals (Figure 5). This finding provided useful clues regarding the function of these genes and may indicate that only the *CYC/TB1* genes in the TCP family are involved in floral zygomorphy in vetch. The three genes in the CYC/TB1 clade observed in vetch also supported the conclusion that most papilionoid legumes exhibit three kinds of *CYC* genes (*LEGCYC IA, IB, and II*), among which the *LEGCYC I* genes experienced a duplication near the base of or below the papilionoid legumes [72].

In vetch, 462 unigenes were found to be specifically expressed in the dorsal petals with the similar expression patterns as the *VsCYC1* and *VsCYC2* genes (Table S7D), indicating that some of the 462 unigenes might be downstream targets of the *VsCYC1* and *VsCYC2* transcription factors, which could be identified based on the presence or absence of putative CYC binding sites in upstream promoter sequences in the 462 unigenes [23]. By the GO term analyses of 462 dorsal petal specific unigenes in vetch (Table S8A) and the identification of the 104 putative CYC or TCP binding sites in 49 homologous genes in *M. truncatula* (Table S8B), our results might offer some important clues to the potentially putative downstream genes of the *CYC* genes in the zygomorphic species. Internal asymmetry is the other important trait associated with zygomorphy, including the internal asymmetry of the lateral and ventral petals in Papilionoideae. A *SYP1* (*SYMMETRIC PETALS 1*) locus was identified from *P. sativum*, whose function is to establish the internal asymmetry of the petals, and *syp1* mutant flowers bearing all symmetric petals without normal internal asymmetry [21]. However, the *SYP1* gene has not been cloned from the *SYP1* locus. In vetch, we found 435 and 922 unigenes that were specifically expressed in the lateral and ventral petals, respectively (Table S7D). These data provided a number of candidate genes that might allow further elucidation of the molecular mechanisms underlying the internal asymmetry in Papilionoideae.

## Conclusions

In the present study, based on *de novo* assembly and RNA-Seq Quantification analysis, we generated genome-wide gene expression profiles for six floral organs in vetch to investigate the characteristics of the evolution and development of zygomorphic flowers. We isolated 71,553 unigenes with an average length of 511 bp and then compared the changes in the expression of these unigenes in the sepals, dorsal petals, lateral petals, ventral petals, stamens, and carpels, using the leaves and roots as controls. By identifying enriched genes in organwise floral categories, our comparison of the gene expression patterns in vetch and *Arabidopsis* revealed that the vetch flower conforms to a strict ABC model, similar to the core eudicots *Arabidopsis* and the basal eudicots *Eschscholzia*, in contrast to the fading transcriptional boundaries observed in the basal eudicots *Aquilegia* [4], [5], [40]. Additionally, regarding the unigenes enriched in the vetch floral organs, most of the homologous transcripts in the counterpart organs in *Arabidopsis* did not display enriched expression, and vice versa. We identified and compared the evolution and expression patterns of the TCP gene family members in vetch at a whole-genome scale, and we further obtained a number of unigenes that were specific to the three different types of vetch petals. These results might provide important clues with respect to elucidating the molecular mechanisms underlying the floral zygomorphy in the Papilionoideae. Our data revealed, for the first time, the comprehensive characteristics of the evolution and development of zygomorphic flowers.

## Supporting Information

Figure S1 **The length distribution of the unigenes obtained using Illumina paired-end technology in vetch.**
(TIFF)

Figure S2 **COG functional classification of the vetch unigenes.**
(TIFF)

Figure S3 **Validation of the expression profiles in the vetch organs.** **a**) Heatmap of the expression levels obtained using Illumina sequencing data and real-time quantitative PCR. The bar represents the scale of the expression levels of the unigenes (log2). *P* denotes the expression detected using real-time PCR, and *S* denotes the expression detected vis Illumina sequencing. Unigene68614 was used as an internal control in the real-time PCR analyses. **b**) Semi-quantitative PCR analysis of 49 unigenes using Unigene68614 as an internal control. Corresponding detailed information is displayed in Table S1.
(TIFF)

Figure S4 **Hierarchical clustering based on the expression patterns in vetch.** Cluster analyses for the vetch floral organs using 71,553 unigenes. The color scale ranges from 0.0 (dark) to 44.0314 (yellow).
(TIFF)

Figure S5 **The distribution of the organ-enriched and downregulated unigenes in vetch with functional annotation.** The genes are classified into 14 functional groups. Positive and negative numbers denote the percentages of unigenes enriched and downregulated in different organs, respectively. Corresponding detailed information is listed in Table S4a and S4b (enriched expression), and S4c and S4d (downregulated expression).
(TIFF)

Figure S6 **Gene Ontology term “enrichment status” for targets of the dorsal petal-specific unigenes.** (**a**–**c**) Targets with GO term “enrichment status” and “hierarchy” for **a**) biological process, **b**) molecular function and **c**) cellular component branches. The classification terms and their serial numbers are represented as boxes. For significant terms, the box includes the GO term, adjusted P-value (in parentheses), item number mapping the GO term in the query list and background, and total number of items in the query list and background. The color scale shows the P-value cutoff levels for each biological process; the more statistically significant, the darker and redder the color.
(TIFF)

Figure S7 **The expression patterns of some key vetch genes.**
(TIFF)

Table S1 **Unigenes examined using quantitative PCR and RT-PCR.**
(XLS)

Table S2 **Correlation coefficients between pairs of floral organs in vetch.**
(XLS)

Table S3 **Statistics for the enriched and the downregulated transcripts in vetch and** ***Arabidopsis*** **.**
(XLS)

Table S4 **Enriched and downregulated unigenes in vetch floral organs and their functional categories.**
(XLS)

Table S5 **Six groups of unigenes enriched in individual organs and combinations of organs in vetch.**
(XLS)

Table S6 **Comparison of enriched transcription factors between vetch and** ***Arabidopsis*** **.**
(XLS)

Table S7 **Homologous comparison of the enriched floral transcripts between vetch and** ***Arabidopsis*** **.**
(XLS)

Table S8 **Homologous comparison between vetch and** ***M. truncatula*** **and** ***cis*** **-elements analyses of** ***M. truncatula*** **.**
(XLS)
