# Supplementary figures and images for: Comparative Transcriptional Profiling Provides Insights into the Evolution and Development of the Zygomorphic Flower of Vicia sativa (Papilionoideae)
Source: PLoS One. 2013 Feb 21;8(2):e57338. doi: 10.1371/journal.pone.0057338 (PMC3578871; doi:10.1371/journal.pone.0057338)

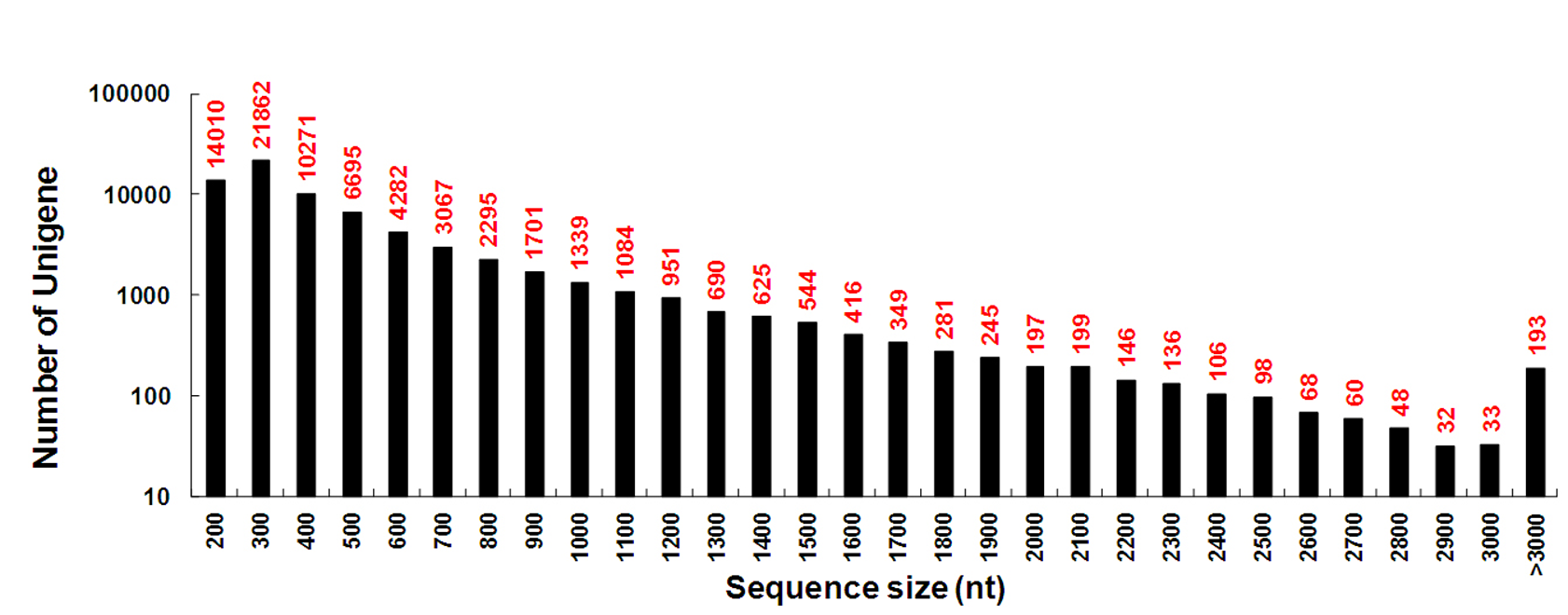

Supplement: Figure S1 — The length distribution of the unigenes obtained using Illumina paired-end technology in vetch. (TIFF) [file pone.0057338.s001.tiff]

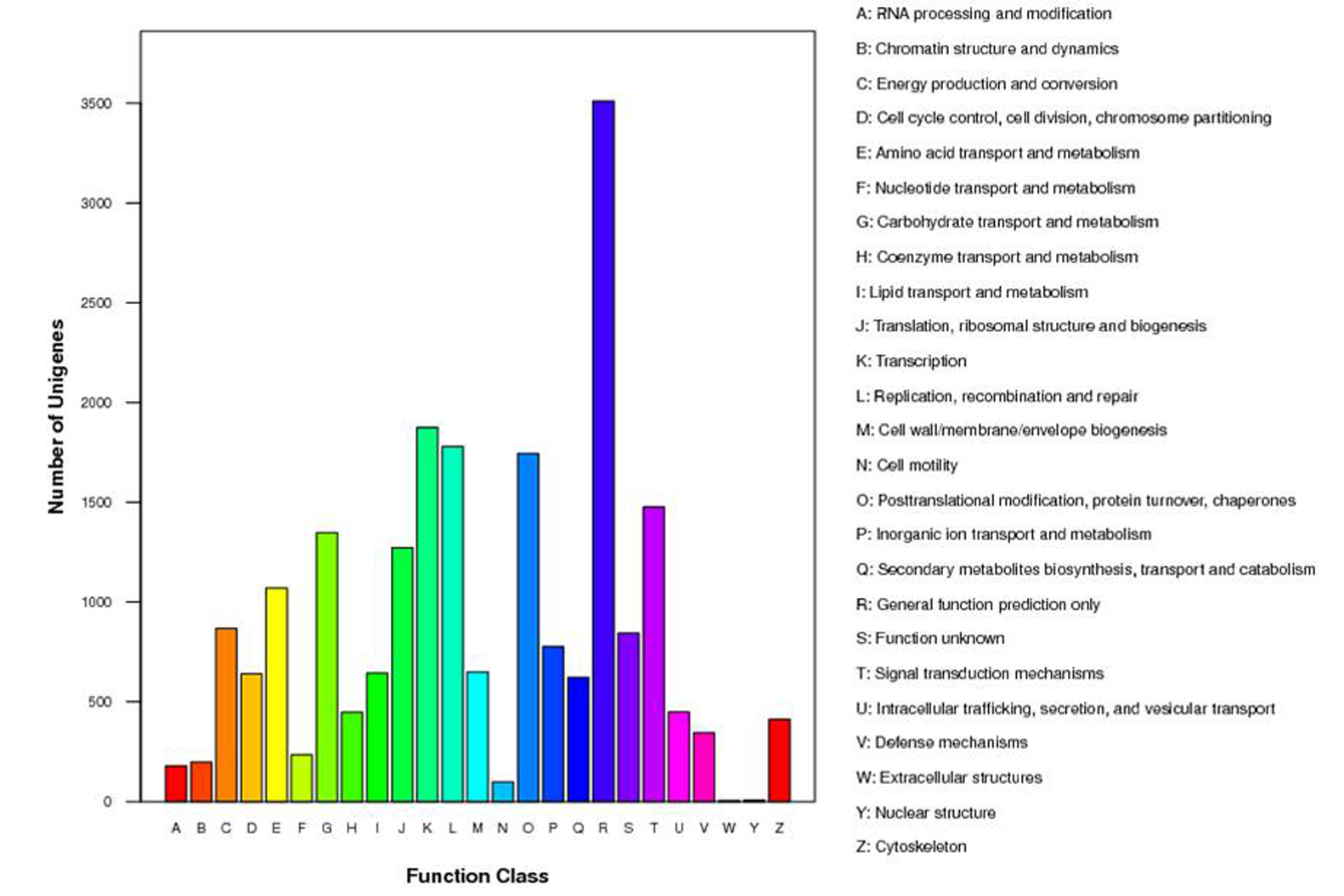

Supplement: Figure S2 — COG functional classification of the vetch unigenes. (TIFF) [file pone.0057338.s002.tiff]

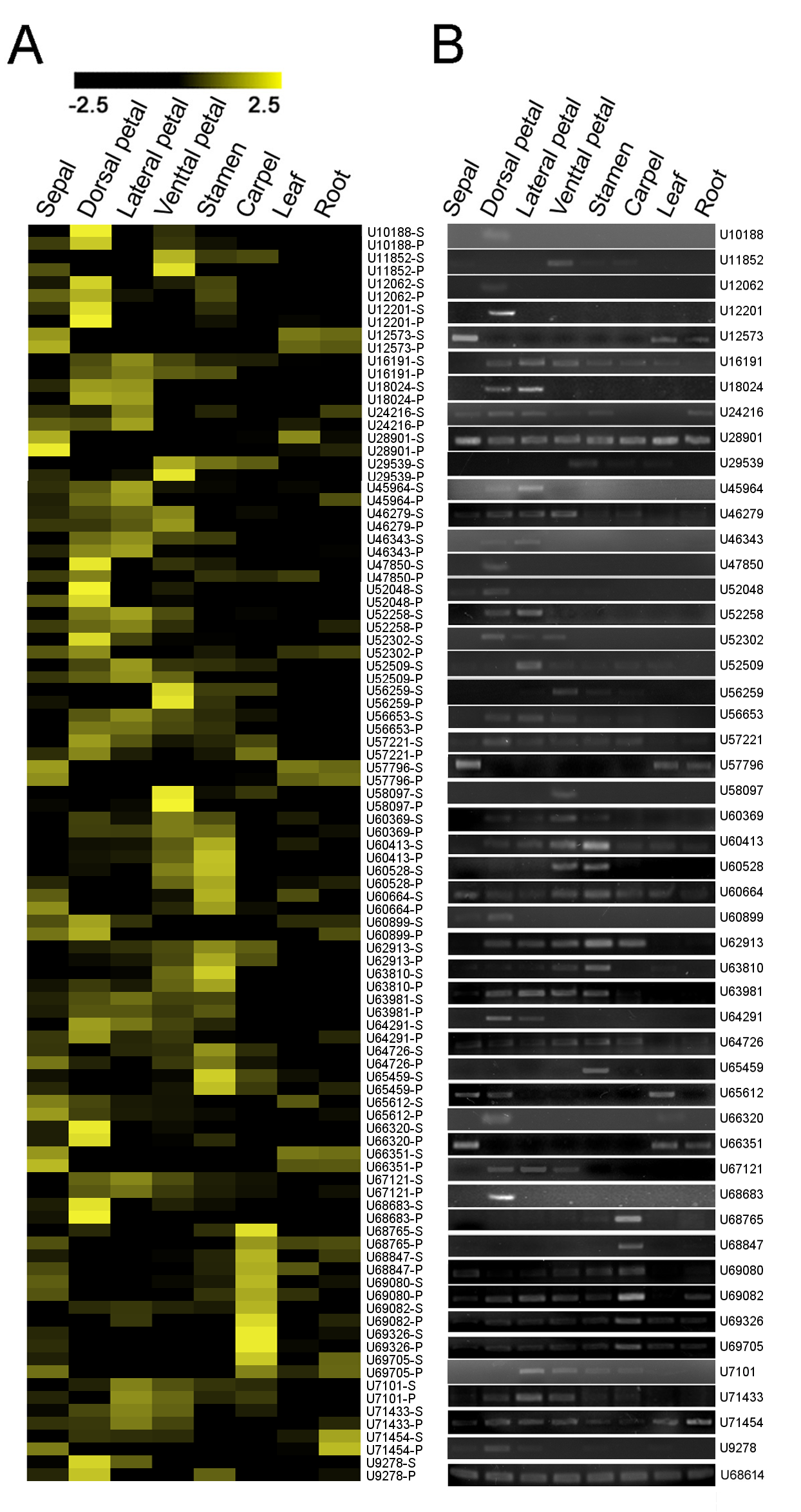

Supplement: Figure S3 — Validation of the expression profiles in the vetch organs. a) Heatmap of the expression levels obtained using Illumina sequencing data and real-time quantitative PCR. The bar represents the scale of the expression levels of the unigenes (log2). P denotes the expression detected using real-time PCR, and S denotes the expression detected vis Illumina sequencing. Unigene68614 was used as an internal control in the real-time PCR analyses. b) Semi-quantitative PCR analysis of 49 unigenes using Unigene68614 as an internal control. Corresponding detailed information is displayed in Table S1. (TIFF) [file pone.0057338.s003.tiff]

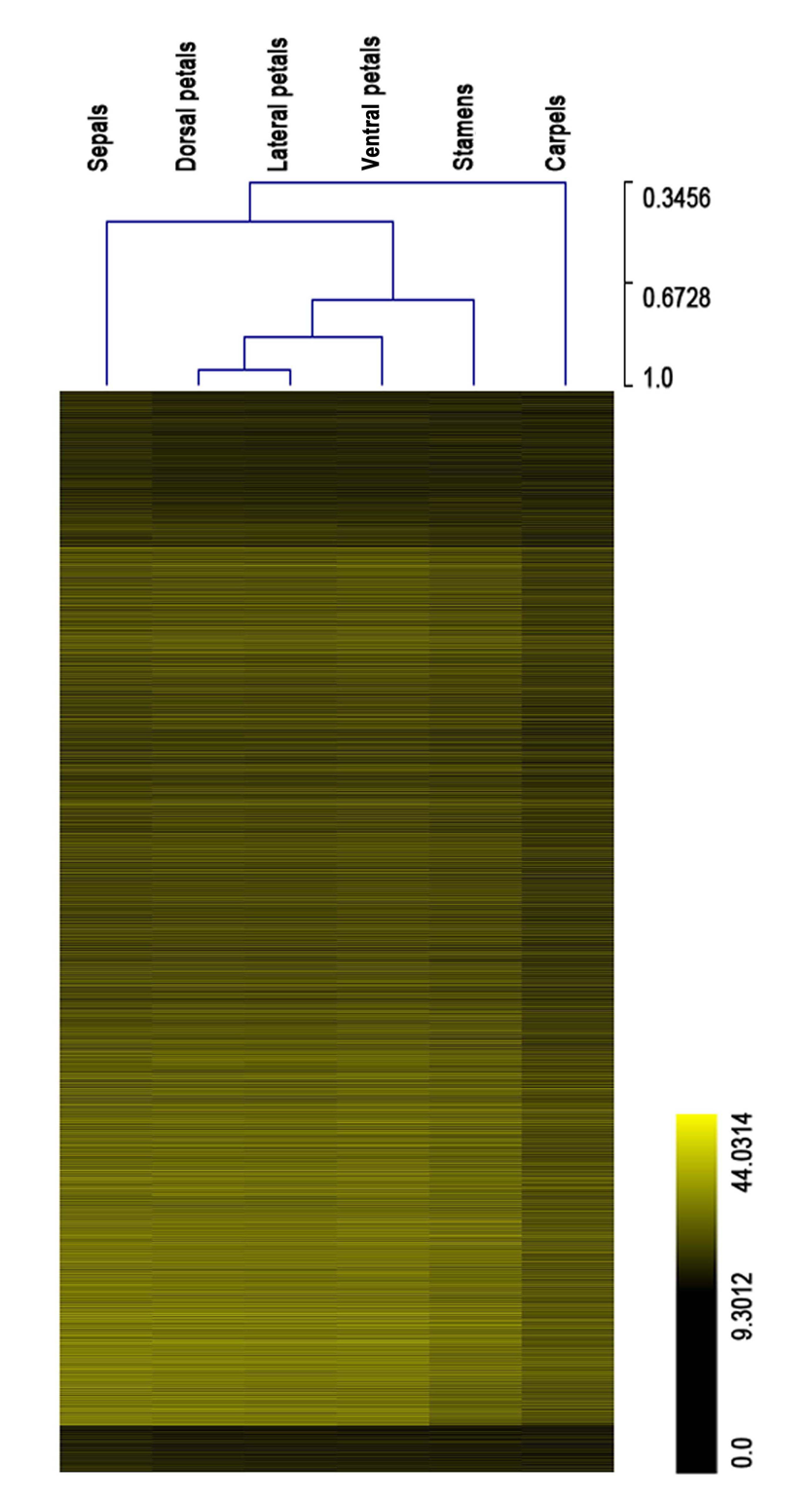

Supplement: Figure S4 — Hierarchical clustering based on the expression patterns in vetch. Cluster analyses for the vetch floral organs using 71,553 unigenes. The color scale ranges from 0.0 (dark) to 44.0314 (yellow). (TIFF) [file pone.0057338.s004.tiff]

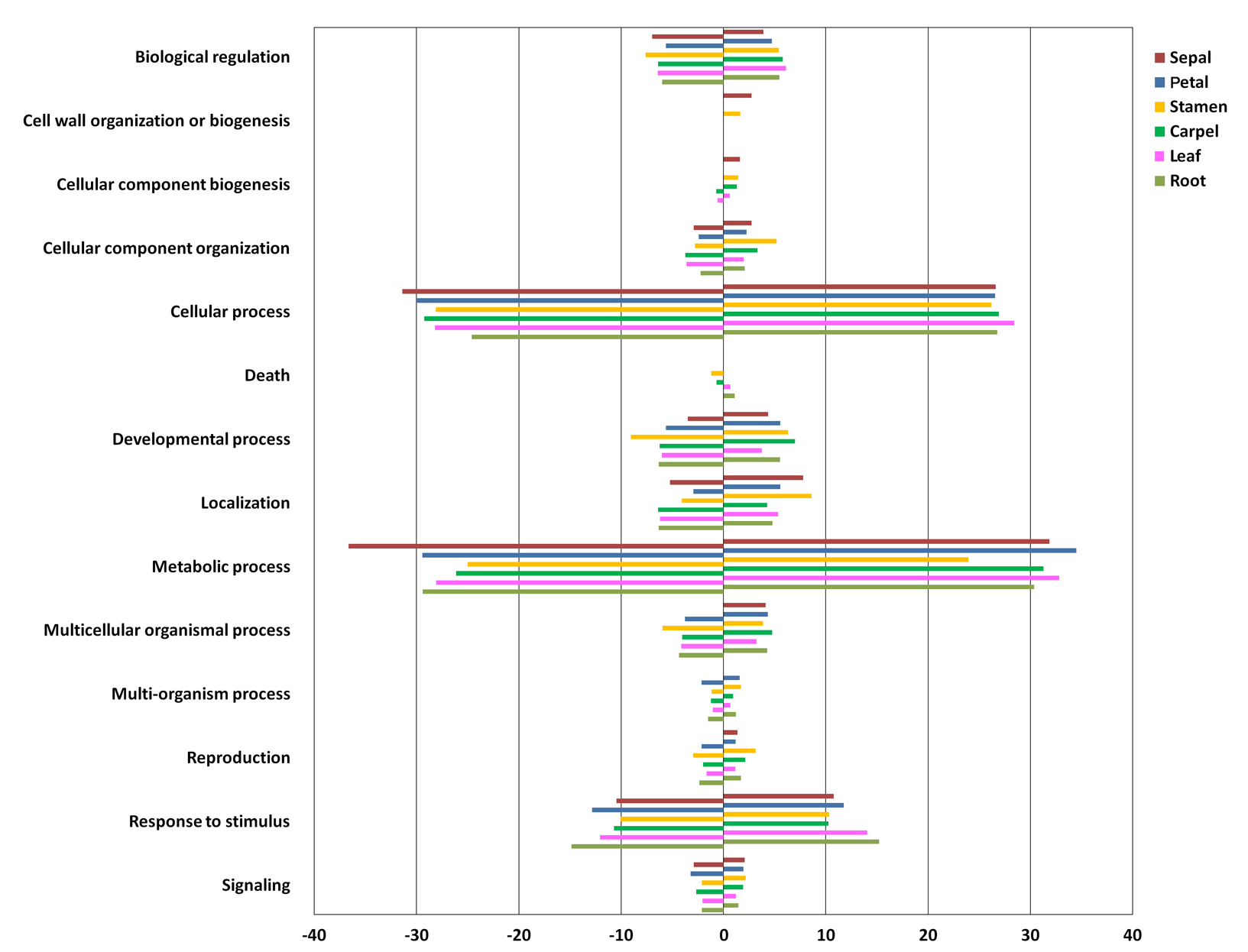

Supplement: Figure S5 — The distribution of the organ-enriched and downregulated unigenes in vetch with functional annotation. The genes are classified into 14 functional groups. Positive and negative numbers denote the percentages of unigenes enriched and downregulated in different organs, respectively. Corresponding detailed information is listed in Table S4a and S4b (enriched expression), and S4c and S4d (downregulated expression). (TIFF) [file pone.0057338.s005.tiff]

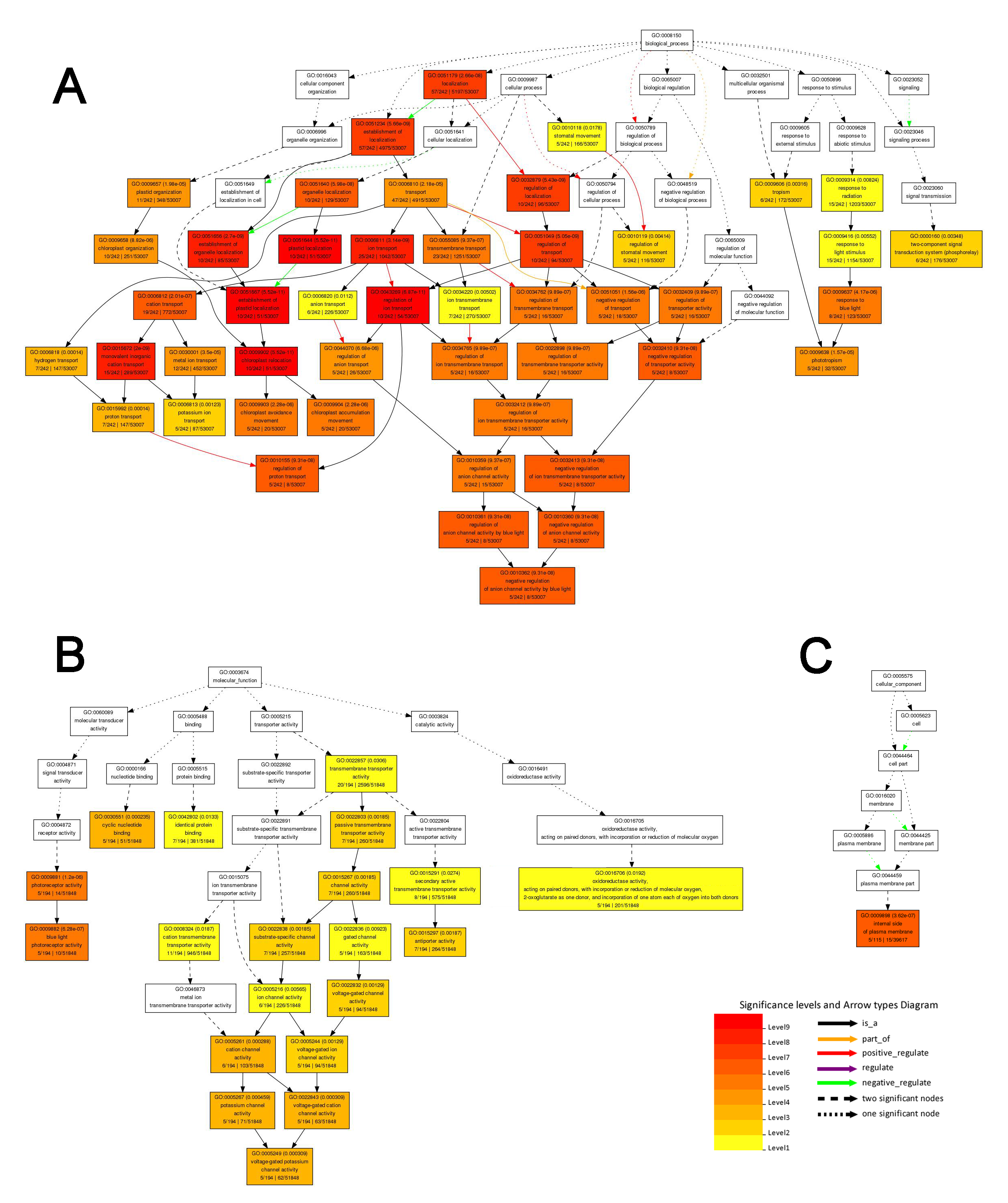

Supplement: Figure S6 — Gene Ontology term “enrichment status” for targets of the dorsal petal-specific unigenes. (a–c) Targets with GO term “enrichment status” and “hierarchy” for a) biological process, b) molecular function and c) cellular component branches. The classification terms and their serial numbers are represented as boxes. For significant terms, the box includes the GO term, adjusted P-value (in parentheses), item number mapping the GO term in the query list and background, and total number of items in the query list and background. The color scale shows the P-value cutoff levels for each biological process; the more statistically significant, the darker and redder the color. (TIFF) [file pone.0057338.s006.tiff]

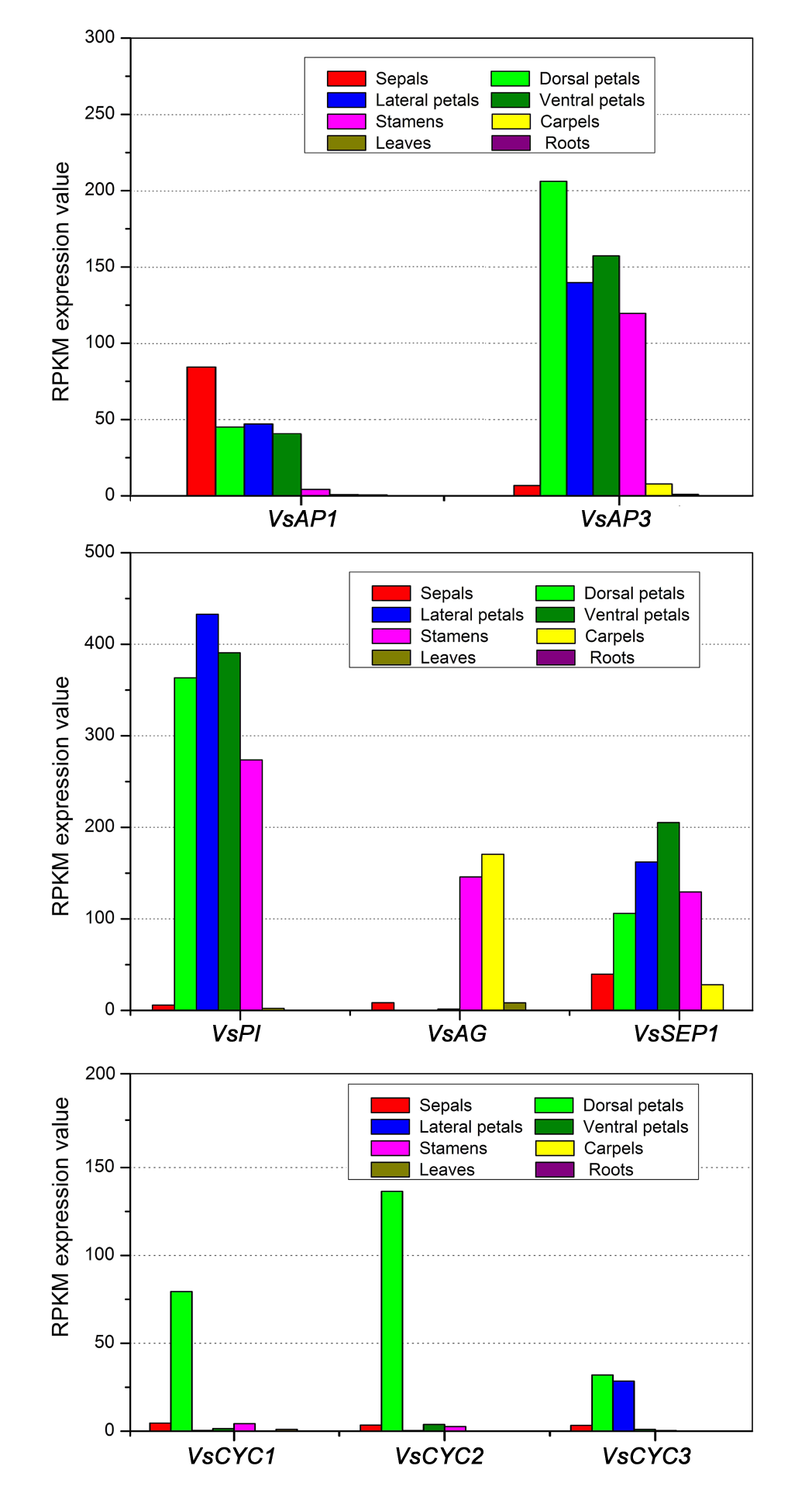

Supplement: Figure S7 — The expression patterns of some key vetch genes. (TIFF) [file pone.0057338.s007.tiff]
